# Gene Expression Changes in the Skin of Patients Undergoing Medial Thigh Liposuction With Pre-Surgical and Post-Surgical Application of Topical Products

**DOI:** 10.1093/asjof/ojaa033

**Published:** 2020-07-03

**Authors:** Mary E Ziegler, Brannon Claytor, Michaela Bell, Laurie Casas, Alan D Widgerow

## Abstract

**Background:**

Skin topical preconditioning before and after surgical procedures is a relatively new concept, particularly in relation to the efficient removal of tissue breakdown products. Clinical trials demonstrate improvements, such as less induration, when surgery is combined with topical product preconditioning and with usage post-surgery.

**Objectives:**

This trial aimed to assess the efficacy of such a regimen at the molecular level through gene expression studies in combination with clinical assessments.

**Methods:**

Six women who underwent medial thigh liposuction administered either a bland moisturizer or the experimental topical products to each side of the surgical area twice daily. Biopsies were taken before any topical application, at 2 and 4 weeks after liposuction. An inflammation-related gene expression analysis was conducted to compare the different conditions. In addition, the degree of induration was assessed in a blinded manner.

**Results:**

Compared with the bland moisturizer, the experimental group demonstrated a hastened immune inflammatory response moving more rapidly to an anti-inflammatory reversal at 2 weeks followed by a wound healing extracellular remodeling effect at 4 weeks. This matched the clinical picture depicting less induration with the treatment.

**Conclusions:**

For patients undergoing body procedures, a topical treatment with the Alastin induces an accelerated healing response, inducing the clearance of “waste” products and the induction of anti-inflammatory genes. Furthermore, this topical treatment stimulates extracellular matrix remodeling, which ultimately leads to less induration.

**Level of Evidence: 5:**

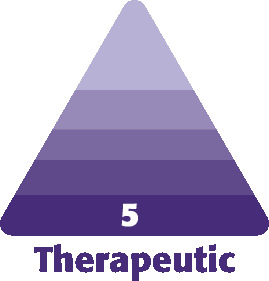

The concept of topically preconditioning the skin before surgery and the maintenance of topical applications post-surgery is attracting much interest. Several clinical observations and biopsy results have demonstrated improvements due to the use of topicals in the peri-procedure period in the skin’s extracellular matrix (ECM) and the resolution of skin induration, fibrous banding, and ecchymosis.^1,2^ This is thought to be due to an improved cross-talk between fibroblasts and collagen and elastin fibers in the ECM as a preconditioning before surgery^2^ coupled with an improvement in macrophage absorption of lipid droplets following surgeries involving the disruption of fat tissue.^3^

From the standpoint of wound healing, for chronic wound management, it is necessary to prepare the wound bed for healing before applying any therapeutics as the buildup of byproducts from dysfunctional healing and excess inflammation can create a wound bed milieu that averts healing. Eliminating these byproducts from the wound bed is a priority in wound bed preparation, encouraging accelerated endogenous healing and facilitating the effectiveness of the therapeutic measures.^4^

Using topical applications to provide “skin bed” preparation has yielded significant improvements based on histological and clinical observations.^1-3^ However, an objective molecular measurement of the changes seen within skin layers that could impact healing and patient comfort is lacking. To this end, a split-body study was designed to compare comprehensive gene expression changes in patients following bilateral extensive medial thigh liposuction with and without the use of a topical treatment.

## METHODS

### Study Population

This was a split-body, randomized, double-blinded study that occurred over 8 ½ months from August 8, 2019 to March 17, 2020. The study was approved by the Integreview IRB (Austin, TX). A total of 6 patients participated in this study. The eligible patients included women undergoing extensive liposuction of the medial thighs. The purpose of selecting liposuction patients was due to the fact that this procedure greatly disrupts the fat, allowing for the determination of whether the treatment improved healing by clearing the debris caused by the fat disruption. The participants agreed to apply the topical products twice daily for 2 weeks before and after surgery. Patients who were not good candidates for the surgery, as determined by the physician, were excluded from participating in the study. Pregnant or lactating patients were excluded as well as those planning to become pregnant during the study duration. This study adhered to the guiding principles of the Declaration of Helsinki, and written informed consent was obtained from all patients. 

### Liposuction Procedure

The liposuction ports were identical in each case. Two incisions on each leg. The superior incision along the bikini line in the upper medial groin and the second along the medial aspect of the thigh 10 cm superior to the medial condyle of the knee. The liposuction access was always remote from the skin biopsy sites. The liposuction volume was consistent at 250 ml per medial thigh with similar suction techniques. 

### Topical Application

The patients were randomized to receive Regenerating Skin Nectar (RSN) with TriHex Technology and TransFORM Body (TFB) treatment with TriHex Technology either on the right or left treatment area. On the opposite side, they received a mild unscented moisturizer (CetaphilLotion, Galderma Laboratories, Fort Worth, TX), which was applied from 2 separate bottles to unsure that the patients were blinded to the experimental condition. The kits and patients numbers were randomized in excel, and the investigator was blinded. The patients were preconditioned with RSN 2 weeks before the elected surgery on the designated side. Immediately after the procedure, they applied RSN and then TFB to the designated side, around the incision and procedure area for up to 10 weeks or longer as determined by the physician. On the opposite side, the patients applied the moisturizer 2 weeks before surgery and after the surgery for up to 10 weeks. Cetaphil was used as a comparative moisturizer. Our goal was to ensure that general moisturizing was not accounting for the effects we observed. The Cetaphil was meant to counter the effects of the increased hydration that was caused by the use of the treatment products.

### Punch Biopsies

Among the 6 patients, 5 consented to punch biopsies (3 mm) in the surgical area of both treatment sides. Three patient biopsies were subjected to gene expression analysis, while the remaining 2 were submitted for histological examination (1 patient refused biopsies). The biopsy sites were located along a line drawn from the insertion of the *adductor brevis* on the inferior ramus of the pubis to the medial condyle of the femur at the knee. The first biopsy was 8 cm inferior to the adductor insertion, the second biopsy was 10 cm, and the third was 12 cm inferior. The biopsies were performed at the pretreatment stage and at weeks 2 and 4 post-surgery. All biopsies were performed by a board-certified plastic surgeon. Upon collection, the biopsies for gene expression were immediately placed in RNAlater (Invitrogen, Carlsbad, CA) stabilization solution and stored overnight at room temperature. Then, the samples were stored at −20°C until they were shipped to Genemarkers (Kalamazoo, MI) for RNA processing. A total of 18 clinical skin biopsies were obtained from 3 patients assigned for gene expression studies.

### RNA Isolation

RNA isolation and subsequent RNA analyses described below were performed by Genemarkers. Briefly, total RNA was isolated from each biopsy using an RNeasy Mini kit (Qiagen, Germantown, MD) following the manufacturer’s instructions for fibrous tissues. RNA concentration and purity were determined using a Nanodrop 2000 spectrophotometer (ThermoFisher, Waltham, MA). RNA integrity was assessed using an Agilent Bioanalyzer 2100 (Santa Clara, CA). All samples showed high-quality RNA metrics and similar yields were obtained for all samples, with the exception of one sample. This sample had a low RNA yield and required vacuum concentration.

### Endogenous Control Gene Selection

An endogenous control gene was selected that was consistently expressed in all of the samples for comparison. The Thermo Fisher (TF) Human Inflammation Panel includes 21 endogenous controls tested across 29 separate assays. Three algorithms (Normfinder algorithm, Minimum Variance Median algorithm [minvarmed], and the coefficient of variability [CV]) were used to calculate the stability scores in order to determine the most consistent endogenous control gene. Lower stability scores represented a more consistent expression between samples in the study. Based on the stability scores and average rankings of the endogenous controls, hypoxanthine phosphoribosyltransferase 1 was identified as the most stable endogenous control gene. 

### Reverse Transcription, Product Preparation, and Preamplification

The TF Human Inflammation Panel contains 586 validated TaqMan Assays related to human inflammation. The reverse transcription (RT) and preamplification steps were carried out in an Applied Biosystems 2720 Thermal Cycler (Foster City, CA), according to the manufacturer’s instructions for Low Sample Input on TaqMan OpenArray Pathway Panels (Life Technologies, Carlsbad, CA). Two separate gene-specific RT products were generated from 100 ng of each RNA sample using a SuperScript Vilo RT Kit (Invitrogen, Carlsbad, CA) and 2 custom Taqman PreAmp Pools (Applied Biosystems), labeled pool A or pool B (10 minutes at 25°C, 60 minutes at 42°C, and 5 minutes at 85°C). Each RT product was then preamplified using Taqman Preamplification Master Mix (Applied Biosystems), the same custom Taqman PreAmp Pool (A or B), and 14 cycles of preamplification (15 seconds at 95°C and 4 minutes at 60°C). At the end of the preamplification, the products from pools A and B for each sample were mixed thoroughly and then diluted 1:20 with nuclease-free water.

### qPCR Processing and Analysis

The qPCR reactions were performed in an OpenArray format in the Life Technologies QuantStudio 12K Flex instrument. Each gene was assayed in duplicate. The qPCR data quality was assessed and exported from the raw data files using Expression Suite software (Applied Biosystems). The qPCR data were then imported into the “OmicsOffice for qPCR” tool of TIBCO Spotfire Analyst software. Statistical data analysis was performed using the relative quantitation (RQ) method. In the first step of an RQ analysis, the cycle threshold (CT) value of the target gene was normalized to the CT value of an endogenous control gene to generate the delta CT (dCT). The dCT values were calculated to normalize the variability between the samples that might occur during the experimental procedures. (See the Endogenous Control Gene Selection section for more details).

### SkinFibroMeter Measurements

The patients were assessed using SkinFibroMeter (Delfin Technologies, Miami, Fl) measurements. The SkinFibroMeter assesses induration in absolute units of stiffness. The patients underwent a pretreatment visit, a baseline (Day 0) visit, and follow-up visits at every week initially, then at 2 weeks, and then every 3 weeks until 10 weeks post-procedure/surgery. An increased value over the baseline reading was indicative of stiffness/induration. The SkinFibroMeter data are presented for the 1-week and 2-week time points for the 6 patients for both the treated and untreated sides. The readings at 4 weeks and beyond leveled out and are not presented.

### Investigator Assessment

To assess induration, the investigators were blinded to the treatments. The level of induration was assessed using a graded scale as follows: 0 is none; 1 is barely perceptible; 2 is slight; 3 is moderate; and 4 is severe. All 6 patients were assessed at the follow-up visits at every week initially, then at 2 weeks, and then every 3 weeks until 10 weeks post-procedure/surgery. The data presented are from the 2-week and 4-week follow-ups, which correspond to the biopsy time points.

### Statistical Analysis

For the gene expression data, paired *t*-tests (*N* = 3, *P* < 0.05) were performed using TIBCO Spotfire software (Palo Alto, CA). The statistical comparison generated delta delta CT (ddCT) values (the mean dCT of the treated group – the mean dCT of the control group). The statistical software converted the ddCT values into log and linear RQ values for export (RQ = 2 − ddCT). The linear RQ values were converted to linear fold-change values to simplify data interpretation. The linear fold-change data were calculated from the exported linear RQ values using Microsoft Excel as follows: for the RQ values > 1.0, the linear fold-change value = RQ value, and for the RQ values < 1.0, the linear fold-change value = −1/RQ value. The percent change was also provided and calculated as the RQ value minus 1. For the clinical assessment, Student’s *t*-tests were performed using Microsoft Excel. *P* < 0.05 was considered significant.

## RESULTS

### Assessing Overall Gene Expression Changes

The biopsies used for gene expression were all from female patients with a mean age of 36 years (range, 33-38 years). The gene expression changes for the 2-week and 4-week biopsies were compared with the pretreatment biopsies for the treated and untreated samples separately. The results are presented in Table 1. The genes included in the table have a change in gene expression ≥1.5-fold in at least one comparison. Based on these results, the gene expression results were analyzed using the different groups as described below.

**Table 1.** Assessing Gene Expression Changes Compared With the Pretreatment Biopsies

Gene-assay	2W vs pre		4W vs pre	
	UT	T	UT	T
AGTR1-Hs00258937_m1	n.s.	2.09	n.s.	n.s.
BDKRB2-Hs00176121_m1	n.s.	n.s.	n.s.	−1.54
BMP7-Hs00233476_m1	−1.61	n.s.	n.s.	n.s.
BMP8A-Hs00257330_s1	n.s.	−1.50	−1.04	n.s.
C1R-Hs00354278_m1	n.s.	2.50	n.s.	1.39
C1S-Hs01043795_m1	3.22	n.s.	n.s.	n.s.
C3-Hs01100879_m1	n.s.	1.73	n.s.	n.s.
CCL17-Hs00171074_m1	2.00	n.s.	n.s.	3.30
CCL19-Hs00171149_m1	n.s.	n.s.	n.s.	3.52
CCL20-Hs01011368_m1	n.s.	3.85	n.s.	3.12
CCL22-Hs00171080_m1	n.s.	n.s.	n.s.	2.19
CCL27-Hs00171157_m1	−2.33	−2.86	n.s.	n.s.
CCRL1-Hs00664347_s1	2.42	n.s.	n.s.	n.s.
CCRL2-Hs00243702_s1	n.s.	1.88	n.s.	1.37
CD40LG-Hs00163934_m1	n.s.	n.s.	n.s.	1.70
CD55-Hs00892618_m1	n.s.	n.s.	n.s.	−1.55
CFD-Hs00157263_m1	1.88	n.s.	n.s.	n.s.
CHRNA7-Hs01063373_m1	2.83	1.41	1.81	n.s.
CLCF1-Hs00757942_m1	n.s.	n.s.	n.s.	−1.63
CLU-Hs00156548_m1	n.s.	1.55	n.s.	n.s.
CRLF1-Hs00191064_m1	−1.91	n.s.	−1.91	−2.05
CYBB-Hs00166163_m1	n.s.	1.64	n.s.	n.s.
EBI3-Hs00194957_m1	n.s.	n.s.	n.s.	2.62
FIGF-Hs01128659_m1	n.s.	3.19	n.s.	n.s.
FLT3LG-Hs00181740_m1	n.s.	n.s.	n.s.	1.86
GDF15-Hs00171132_m1	n.s.	n.s.	n.s.	2.47
HGF-Hs00300159_m1	2.48	n.s.	n.s.	n.s.
IL11-Hs00174148_m1	n.s.	n.s.	2.06	n.s.
IL1F9-Hs00219742_m1	n.s.	n.s.	−1.66	−1.79
IL21R-Hs00222310_m1	n.s.	1.57	n.s.	1.57
IL27RA-Hs00945029_m1	n.s.	n.s.	n.s.	1.52
IL32-Hs00170403_m1	n.s.	n.s.	n.s.	1.70
IL33-Hs01125943_m1	2.53	n.s.	n.s.	n.s.
IL3RA-Hs00608141_m1	1.56	n.s.	n.s.	n.s.
IL6-Hs00985639_m1	1.94	3.34	n.s.	n.s.
IL9R-Hs01108522_m1	n.s.	−1.61	n.s.	n.s.
ITGB2-Hs00164957_m1	n.s.	2.84	n.s.	n.s.
KLRG1-Hs00929964_m1	n.s.	2.18	n.s.	n.s.
LEFTY1-Hs00764128_s1	−1.87	n.s.	n.s.	n.s.
LEPR-Hs00174497_m1	n.s.	−2.13	n.s.	n.s.
LTBP4-Hs00186025_m1	n.s.	n.s.	n.s.	−1.52
LTC4S-Hs00168529_m1	n.s.	1.55	n.s.	n.s.
LY86-Hs00169454_m1	n.s.	2.03	n.s.	1.66
MMP25-Hs01554789_m1	n.s.	n.s.	n.s.	1.52
NFAM1-Hs00377608_m1	n.s.	1.80	n.s.	n.s.
NFATC4-Hs00190037_m1	n.s.	1.95	n.s.	n.s.
NLRP12-Hs00536435_m1	n.s.	n.s.	n.s.	2.29
NLRP3-Hs00918082_m1	n.s.	3.15	n.s.	n.s.
PLA2G4C-Hs00234345_m1	2.19	1.73	n.s.	n.s.
PLCB2-Hs00190117_m1	n.s.	3.30	n.s.	2.21
SCUBE1-Hs00542698_m1	n.s.	n.s.	n.s.	2.57
SELE-Hs00950401_m1	n.s.	n.s.	−2.06	n.s.
SERPINA1-Hs01097800_m1	n.s.	2.96	n.s.	n.s.
SIGIRR-Hs00222347_m1	1.64	n.s.	n.s.	n.s.
SIGLEC1-Hs00988063_m1	n.s.	2.39	n.s.	n.s.
SLPI-Hs00268204_m1	−1.51	n.s.	n.s.	n.s.
SPN-Hs00174604_m1	n.s.	n.s.	n.s.	1.54
TACR1-Hs00185530_m1	1.78	n.s.	n.s.	n.s.
TGM2-Hs00190278_m1	n.s.	n.s.	n.s.	4.26
TNFRSF4-Hs00533968_m1	2.09	1.94	n.s.	n.s.
TNFSF13B-Hs00198106_m1	n.s.	2.33	n.s.	n.s.
TNFSF15-Hs00270802_s1	1.73	2.06	n.s.	n.s.
TPST1-Hs00234324_m1	2.06	1.88	n.s.	n.s.

All of the values are the fold change relative to the pretreatment biopsy samples. Fold changes ≥1.5 were used for further analyses. 2W vs pre, biopsy results at 2 weeks post surgery compared to pre-surgical biopsy; 4W vs pre, biopsy results at 4 weeks post surgery compared to pre-surgical biopsy; CCL, chemokine ligand; CYBB, cytochrome b beta chain; HGF, hepatocyte growth factor; NLRP, Nucleotide-binding oligomerization domain, Leucine rich Repeat and Pyrin domain containing; n.s., not significant; TGM, transglutaminase; T, treated; UT, untreated.

### Comparison of the Biopsies Collected at 2 Weeks From the Untreated and Treated Groups

**Figure 1.**
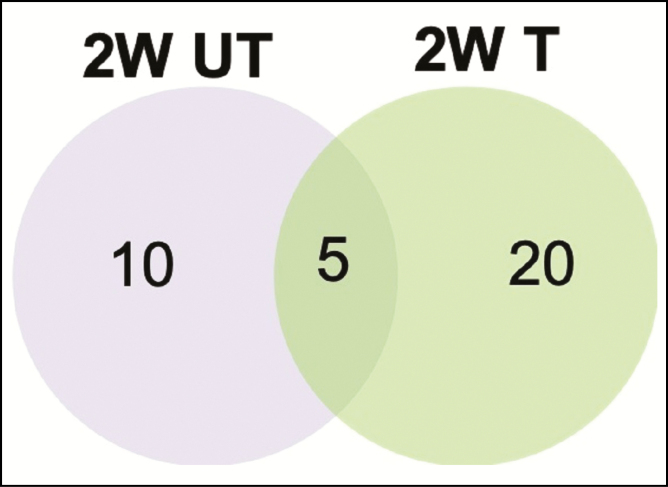
Venn diagram comparing the upregulated genes in the 2-week untreated vs treated groups. The numbers are based on the genes that were significantly upregulated (≥1.5-fold) in comparison to the pretreatment biopsies. The data are based on Table 1. 2W UT, 2-week-untreated group; 2W T, 2-week-treated group.

To assess how the treatment affected healing after 2 weeks, the gene expression data from the biopsies collected at 2 weeks from the untreated and treated groups were analyzed. Among the significantly upregulated genes in each group, 5 genes were shared between the untreated and treated groups (Figure 1). A closer examination of the fold-change differences between the 5 genes for the treated and untreated groups revealed that the biggest fold-change difference between the groups was for the gene interleukin (IL)-6. This difference was 1.94-fold for the untreated group and 3.34-fold for the treated group (Table 2).

**Table 2.** Common Genes Between the 2-Week Untreated and Treated Groups

Gene	2-Week-untreated group	2-Week-treated group
IL6	1.94	3.34
PLA2G4C	2.19	1.73
TNFRSF4	2.09	1.94
TNFSF15	1.73	2.06
TPST1	2.06	1.88

All of the values are the fold change relative to the pretreatment biopsy samples.

Next, we assessed the upregulated genes from the 2 groups using the String database (https://string-db.org/).^5^ STRING is a database of known and predicted protein–protein interactions. The interactions are both direct and indirect associations that are derived from computational prediction, knowledge transfer between organisms, and from interactions aggregated from other (primary) databases. Gene ontology (GO) annotations are also imported in order infer interactions and report enrichments.

**Figure 2.**
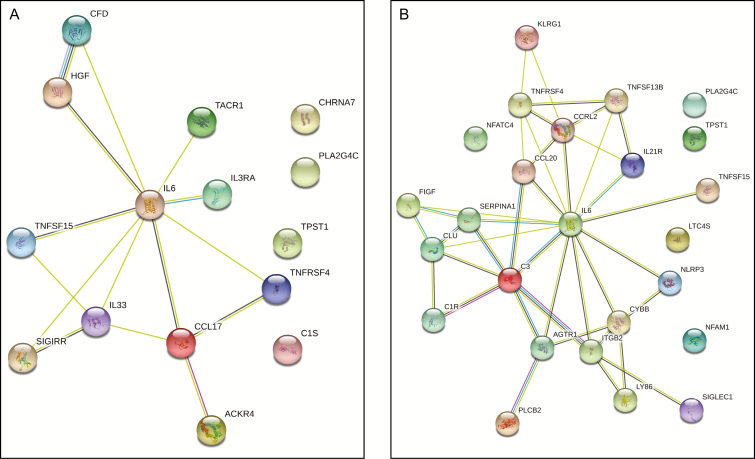
Protein–protein interaction networks for the 2-week groups. The String database was used to compare the interaction networks for the (A) 2-week-untreated group and the (B) 2-week-treated group.

The upregulated genes in the untreated and treated groups from the 2-week biopsy samples were both centered around IL-6 (Figure 2). However, the protein–protein interactions beyond IL-6 were very distinct. This is explained through the GO analysis, which revealed that each group had a discrete enrichment of GO terms. Given that our gene expression analysis was biased because it only looked at inflammatory-related genes, it is not surprising that inflammation was the key signature of the GO analysis. However, because the 2 groups shared only 5 upregulated genes in common, this indicated that the healing processes were different related to the topical treatments. As indicated in Table 3, the untreated group showed a strong pro-inflammatory response, which was expected as a reaction to the surgical procedure. However, in the treated group, the inflammatory reaction was no longer solely a pro-inflammatory response. The GO term analysis revealed the activation of the adaptive immune system as well as the effector response, indicating the activation of clearance (Table 3).

**Table 3.** Unique Gene Ontology Results from the 2-Week Untreated vs Treated Group Comparison

2-Week-untreated group		2-Week-treated group	
	Biological process		Biological process
GO:0071345	Cellular response to cytokine stimulus	GO:0032940	Secretion by cell
GO:0001818	Negative regulation of cytokine production	GO:0002443	Leukocyte mediated immunity
GO:0032675	Regulation of interleukin-6 production	GO:0051716	Cellular response to stimulus
GO:0045079	Negative regulation of chemokine biosynthetic process	GO:0045087	Innate immune response
GO:0002526	Acute inflammatory response	GO:0032103	Positive regulation of response to external stimulus
GO:0032642	Regulation of chemokine production	GO:0002699	Positive regulation of immune effector process
GO:0045778	Positive regulation of ossification	GO:0002824	Positive regulation of adaptive immune response
GO:0001976	Neurological system process involved in regulation of systemic arterial blood pressure	GO:0070887	Cellular response to chemical stimulus
GO:0002830	Positive regulation of type 2 immune response	GO:0002705	Positive regulation of leukocyte mediated immunity
GO:0033135	Regulation of peptidyl-serine phosphorylation	GO:0006958	Complement activation, classical pathway
	Molecular function		Molecular function
GO:0004896	Cytokine receptor activity	GO:0005102	Signaling receptor binding
		GO:0001664	G protein-coupled receptor binding
		GO:0005164	Tumor necrosis factor receptor binding
		GO:0048020	CCR chemokine receptor binding
		GO:0005515	Protein binding
		GO:0038023	Signaling receptor activity
		GO:0001540	Amyloid-beta binding
		GO:0005488	Binding
		GO:0046982	Protein heterodimerization activity
		GO:0046983	Protein dimerization activity
	Cellular component	Cellular component	
	None	GO:0005615	Extracellular space
		GO:0005576	Extracellular region
		GO:0030141	Secretory granule
		GO:0031093	Platelet alpha granule lumen
		GO:0016020	Membrane
		GO:0071944	Cell periphery
		GO:0034774	Secretory granule lumen
		GO:0005783	Endoplasmic reticulum
		GO:0005886	Plasma membrane
		GO:0016021	Integral component of membrane
		GO:0044433	Cytoplasmic vesicle part
		GO:0005887	Integral component of plasma membrane
		GO:0012505	Endomembrane system
		GO:0070821	Tertiary granule membrane
		GO:0044432	Endoplasmic reticulum part
		GO:0044425	Membrane part
		GO:0035579	Specific granule membrane
		GO:0005788	Endoplasmic reticulum lumen
		GO:0030667	Secretory granule membrane

Furthermore, in terms of molecular function, the untreated group revealed cytokine receptor activity, whereas the treated group showed activation of immune modulation (Table 3). For the cellular component GO terms, the untreated group showed no enrichment, whereas the treated group showed a strong enrichment for terms related to the plasma membrane, extracellular space, and secretory vessels. Overall, the GO analysis revealed that the untreated group displayed an immune response that was typical after a surgical procedure, while the treatment group had an altered immune response in a manner that promoted clearance and healing.

Finally, we examined reactome pathways (https://reactome.org/) that were enriched in the groups after 2 weeks. The untreated group showed reactome pathways related to mitogen and interleukin stimulation as well as PI3K/Akt activation (Table 4). Together, these pathways are considered pro-inflammatory regulators. In contrast, the unique pathways enriched in the treated group triggered anti-inflammatory, immunomodulatory, and clearance responses. Taken together, the biopsies collected at 2 weeks from the treated and untreated sides showed strikingly different patterns of gene expression. The untreated side revealed typical inflammatory signaling that would be expected in response to the procedure. In contrast, the treated side revealed that the inflammatory response favored pro-clearance and preparation for healing.

**Table 4.** Unique Reactome Pathway Results from the 2-Week Untreated vs Treated Group Comparison

2-Week-untreated group		2-Week-treated group	
	Reactome pathway		Reactome pathway
HSA-449147	Signaling by interleukins	HSA-168249	Innate immune system
HSA-5684996	MAPK1/MAPK3 signaling	HSA-198933	Immunoregulatory interactions between a lymphoid and a nonlymphoid cell
HSA-6811558	PI5P, PP2A, and IER3 regulate PI3K/AKT signaling	HSA-977606	Regulation of Complement cascade
HSA-446652	Interleukin-1 family signaling	HSA-375276	Peptide ligand-binding receptors
		HSA-6798695	Neutrophil degranulation
		HSA-8957275	Post-translational protein phosphorylation
		HSA-381426	Regulation of insulin-like growth factor (IGF) transport and uptake by insulin-like growth factor-binding proteins (IGFBPs)
		HSA-6783783	Interleukin-10 signaling
		HSA-380108	Chemokine receptors bind chemokines
		HSA-1280218	Adaptive immune system
		HSA-449147	Signaling by interleukins
		HSA-109582	Hemostasis
		HSA-194138	Signaling by VEGF

### Comparison of the Biopsies Collected at 2 Weeks and 4 Weeks From the Treated Group

**Figure 3.**
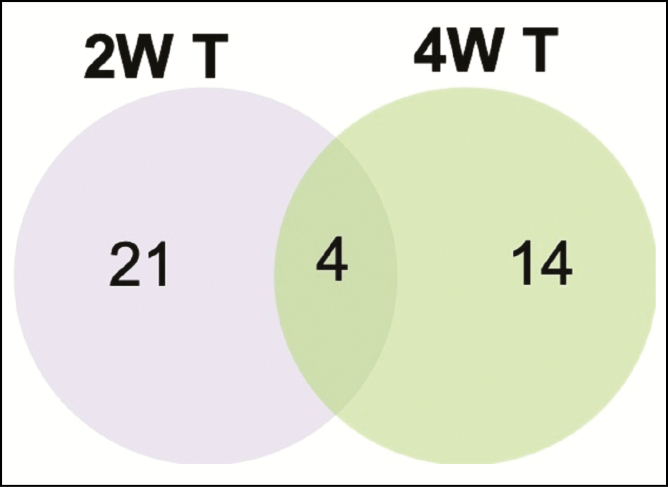
Venn diagram comparing the upregulated genes in the 2-week-treated vs the 4-week-treated groups. The numbers are based on the genes that were significantly upregulated (≥1.5-fold) in comparison to the pretreatment biopsies. The data are based on Table 1. 2W T, 2-week-treated group; 4W T, 4-week-treated group.

The gene expression data from the biopsy samples collected from the 4-week-untreated group revealed that only 2 genes (*CHRNA7* and *IL-11*) were differentially upregulated in comparison to the pretreatment sample. These results indicated that by 4 weeks, the untreated group had essentially returned to baseline. However, in the 4-week-treated group, 18 genes were significantly upregulated compared with the pretreatment group. Given this observation, our next comparison was between the 2-week- and 4-week-treated groups, with the goal of understanding more about what the treatment was doing to further activate the healing patterns that were displayed at 2 weeks. Among the significantly upregulated genes in the 2-week- and 4-week-treated groups (Table 1), there were only 4 genes in common, including chemokine ligand (*CCL20*), *IL21R, LY86*, and *PCLB2* (Figure 3). All of these genes showed a pattern of decreased expression between 2 and 4 weeks, with the exception of *IL21R*, which remained the same (Table 1).

**Figure 4.**
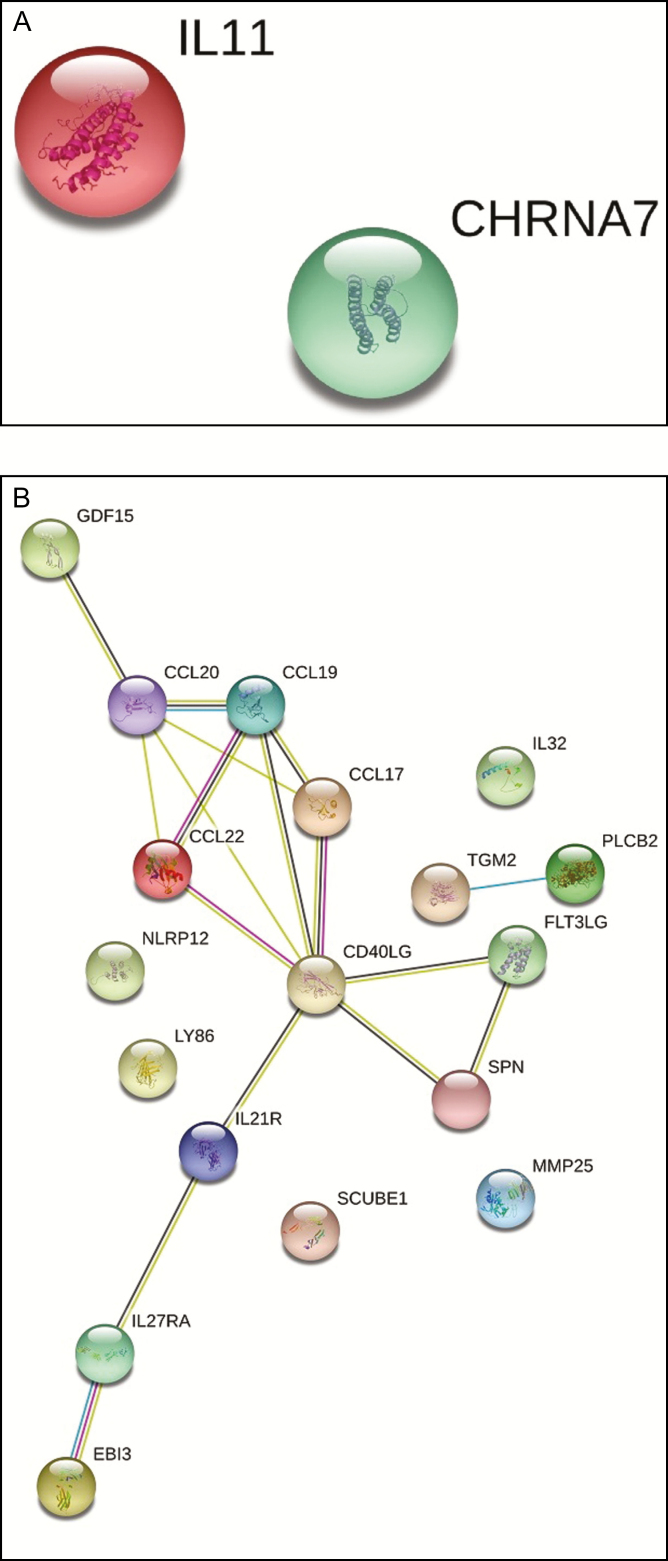
Protein–protein interaction network for the 4-week groups. The String database was used to compare the interaction networks for the (A) 4-week-untreated group and the (B) 4-week-treated group.

The String Database analysis of the 4-week-treated biopsy samples revealed protein–protein interactions that centered around CD40LG (Figure 4).^5^To compare these results to the 2-week-treated biopsy group, the top 10 biological process terms from each group are provided in Table 5. As mentioned above, the 2-week-treated group showed signs of immunomodulatory action based on the unique biological process terms. This was also true for the 4-week-treated group, but there was also an enrichment for terms related to rebuilding and remodeling after injury. For molecular function, the terms were similar but were decreasing with the time of treatment. For the cellular component terms, again, there was a decrease in the number of terms over time, indicating more stability in the tissue (Table 5).

**Table 5.** Unique Gene Ontology Results from the 2-Week-Treated vs the 4-Week-Treated Group Comparison

2-Week-treated group		4-Week-treated group	
#term id	Biological process	#term id	Biological process
GO:0006950	Response to stress	GO:0071345	Cellular response to cytokine stimulus
GO:0002673	Regulation of acute inflammatory response	GO:0048247	Lymphocyte chemotaxis
GO:0032940	Secretion by cell	GO:0048583	Regulation of response to stimulus
GO:0032101	Regulation of response to external stimulus	GO:0050900	Leukocyte migration
GO:0050727	Regulation of inflammatory response	GO:0050670	Regulation of lymphocyte proliferation
GO:0050778	Positive regulation of immune response	GO:1902533	Positive regulation of intracellular signal transduction
GO:0002443	Leukocyte mediated immunity	GO:0031640	Killing of cells of other organism
GO:0051716	Cellular response to stimulus	GO:0043408	Regulation of mapk cascade
GO :0002252	Immune effector process	GO:0061844	Antimicrobial humoral immune response mediated by antimicrobial peptide
GO:0045765	Regulation of angiogenesis	GO:0070372	Regulation of erk1 and erk2 cascade
	Molecular function		Molecular function
GO:0005102	Signaling receptor binding	GO:0008009	Chemokine activity
GO:0048018	Receptor ligand activity	GO:0004896	Cytokine receptor activity
GO:0001664	G protein-coupled receptor binding		
GO:0005164	Tumor necrosis factor receptor binding		
GO:0038023	Signaling receptor activity		
GO:0001540	Amyloid-beta binding		
GO:0005488	Binding		
GO:0004888	Transmembrane signaling receptor activity		
GO:0046982	Protein heterodimerization activity		
GO:0046983	Protein dimerization activity		
	Cellular component		Cellular component
GO:0005576	Extracellular region	GO:0044421	Extracellular region part
GO:0030141	Secretory granule	GO:0009897	External side of plasma membrane
GO:0031093	Platelet alpha granule lumen	GO:0009986	Cell surface
GO:0016020	Membrane		
GO:0071944	Cell periphery		
GO:0034774	Secretory granule lumen		
GO:0005783	Endoplasmic reticulum		
GO:0005886	Plasma membrane		
GO:0016021	Integral component of membrane		
GO:0044433	Cytoplasmic vesicle part		
GO:0005887	Integral component of plasma membrane		
GO:0012505	Endomembrane system		
GO:0070821	Tertiary granule membrane		
GO:0044432	Endoplasmic reticulum part		
GO:0044425	Membrane part		
GO:0035579	Specific granule membrane		
GO:0005788	Endoplasmic reticulum lumen		
GO:0030667	Secretory granule membrane		

Finally, we examined the reactome pathways that were enriched in the 2 treated groups (Table 6). The unique pathways in the 2-week-treated group showed evidence of the activation of anti-inflammatory, immunomodulatory, and clearance signaling processes. In contrast, the 4 unique pathways enriched in the 4-week-treated group were related to not only anti-inflammatory signaling but also macrophage regulation and ECM remodeling. Together, these data provide evidence that the treatment initially activated a gene signature that stimulated clearance and protected the tissue from harm. As time went on, the treatment abolished the pro-inflammatory signature and stabilized an anti-inflammatory environment for the promotion of new ECM and further healing.

**Table 6.** Unique Reactome Pathway Results from the 2-Week-Treated vs the 4-Week-Treated Group Comparison

2-Week-treated group		4-Week-treated group	
	Reactome pathways		Reactome pathways
HSA-168249	Innate immune system	HSA-8984722	Interleukin-35 signaling
HSA-5669034	TNFs bind their physiological receptors	HSA-9020956	Interleukin-27 signaling
HSA-198933	Immunoregulatory interactions between a lymphoid and a nonlymphoid cell	HSA-418594	G alpha (i) signaling events
HSA-977606	Regulation of complement cascade	HSA-1474228	Degradation of the extracellular matrix
HSA-375276	Peptide ligand-binding receptors		
HSA-6798695	Neutrophil degranulation		
HSA-166663	Initial triggering of complement		
HSA-8957275	Post-translational protein phosphorylation		
HSA-114608	Platelet degranulation		
HSA-381426	Regulation of insulin-like growth factor (IGF) transport and uptake by insulin-like growth factor-binding proteins (IGFBPs)		
HSA-1280218	Adaptive immune system		
HSA-109582	Hemostasis		
HSA-194138	Signaling by VEGF		
HSA-6785807	Interleukin-4 and interleukin-13 signaling		

### Induration Assessment

**Figure 5.**
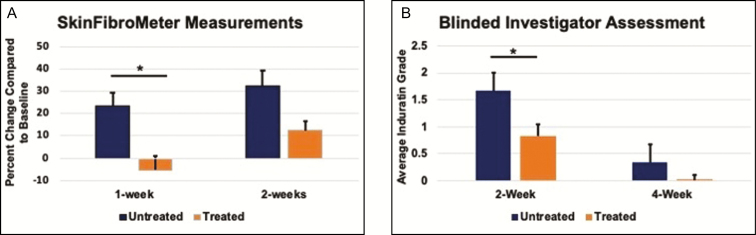
The treatment leads to less induration. (A) The patients were assessed using a SkinFibroMeter, which assesses induration in absolute units of stiffness. An increased value over the baseline reading is indicative of stiffness/induration. The data are presented at the percent increase over baseline for the 1-week and 2-week time points for both the treated and untreated sides. The data are represented as the mean ± standard error the mean. The * indicates *P* < 0.05 (*n* = 6). (B) A blinded investigator assessment was used to assess induration. The level of induration was assessed using a graded scale as follows: 0 is none; 1 is barely perceptible; 2 is slight; 3 is moderate; and 4 is severe. The data represented are the mean ± standard error of the mean from the 2-week and 4-week follow-ups, which correspond to the biopsy time points. The * indicates *P* < 0.05 (*n* = 6).

To assess induration, first, the SkinFibroMeter readings at 1 week and 2 weeks after the procedure for the treated and untreated sides were compared with the baseline (before the procedure) reading. Compared with the baseline reading, the 1-week-untreated group showed a 23% increase, which was a sign of more induration, whereas the 1-week-treated group decreased by 5% compared with the baseline, indicating a lower level of induration. These differences were statistically significant (*P* < 0.05) (Figure 5A). Two weeks after the procedure, the untreated group showed a 32% increase, while the treated group increased by only 12%. These differences were not statistically significant. Taken together, the treated group showed less induration compared with the untreated group.

To further evaluate induration, we used blinded investigator assessments. The data from the 2 and the 4 weeks groups were compared between the treatment groups. At 2 weeks, there was a statistically significant lower induration grade for the treated group. By 4 weeks, the grade was lower for both groups but was approximately 0 for the treated group (Figure 5B). This time point was not statistically significant. These data support that the treatment results in a lower level of induration after the procedure.

## DISCUSSION

Normal everyday living expose patients’ skin to extrinsic damage, mainly in the form of photodamage, which causes ongoing changes within the ECM.^6^Surgical procedures also violate cellular and extracellular structures that impact healing and recovery, which manifest in skin changes. In both these scenarios, one common issue is “waste product” accumulation. In the ECM, waste takes the form of fragmented collagen, elastin, and glycation end products, and can lead to impaired cellular recycling mechanisms involving the proteasome and autophagic processes.^7^ In surgical body contouring procedures, the destruction of fatty tissue releases lipid droplets from the adipose cells, which are highly inflammatory and create localized pockets of “inflammasomes” that can present as skin induration, hardened fibrous banding, and even fat necrosis, where phagocytic processes are overwhelmed. In an effort to deal with these waste products and to optimize their elimination, certain peptides and active agents have been incorporated into topical preparations (RSN with TriHex Technology and TFB Treatment with TriHex Technology, Alastin Skincare Inc., Carlsbad, CA) to improve autophagic processes and macrophage efficiency.^3^ The mechanism of action of these agents have been tested through in vitro assays and clinical studies.^1-4^ However, further objective validation was sought in surgical cases in an effort to merge the clinical impressions with the changes taking place at a molecular level through an examination of gene expression in real-time.

Here, we evaluated the inflammatory gene expression profiles of skin biopsy samples from patients before and after liposuction from areas of the skin that were treated with different topical preparations. Biopsies were taken from the pretreated skin and from the treated and untreated areas at 2 and 4 weeks after the procedure. All gene expression comparisons were made in relation to the pretreatment biopsy. Expression changes predominantly included upregulated genes among the groups.

IL-6 is a pleiotropic regulator of inflammation and immunity.^8^ Its expression initially functions as a warning signal to the entire body in the event of tissue damage.^9^ In comparison to the pretreatment biopsy, both the 2-week-treated and untreated biopsies showed an upregulation of IL-6, which was at the center of their respective protein–protein interaction networks. However, the expression of IL-6 in the treated biopsies at 2 weeks was 1.7-fold higher than that in the untreated group, suggesting that the treatment may produce a different response with respect to IL-6.

The untreated group at 2 weeks revealed that IL-6 interacted with CCL17, IL-33, and IL3RA. These interactions are indicative of the regulation of T regulatory cells,^10,11^ the polarization of a Th2 response to activate macrophages,^12,13^ and the modulation of leukocyte production, proliferation, and survival, which work to enhance acute inflammation.^14^ Furthermore, the network analysis showed an interaction between IL-6 and HGF. HGF balances the inflammatory action caused by the acute phase response by suppressing it.^15^ Together, these gene interactions demonstrated that the 2-week-untreated group displayed a gene pattern that followed the typical inflammatory cascade after surgery, with an enhanced pro-inflammatory signal and the marking of the beginning stages of anti-inflammatory modulation, providing an excellent comparison for the treatment group.

In the 2-week-treated samples, IL-6 showed interactions with IL21R, CCL20, CCRL2, and C3. These connections trigger Th17 cell differentiation,^16^ T and B cell differentiation,^17^ and the regulation of immune tolerance.^18^ In addition, the activation of both innate and adaptive immune responses are part of these pathways^19^ as well as local protection against invading agents.^20^ Thus, compared with the untreated group, these gene expression patterns revealed that the immune response at 2 weeks in the treated group shifted toward an immunomodulatory pattern, indicating a possible anti-inflammatory and M2 macrophage activation profile. Thus, the treatment appeared to have initiated the start of debris clean up at this time point.

This was further substantiated by the interaction of IL-6 with NLRP3 (Nucleotide-binding oligomerization domain, Leucine rich Repeat and Pyrin domain containing) and cytochrome b beta chain in the 2-week-treated group. The NLRP3 inflammasome is activated in primary macrophages and acts as a broad sensor of cell homeostasis rupture. Upon activation, NLRP3 assembles a multiprotein platform that leads to caspase-1 activation, which controls, by direct cleavage, the maturation of cytosolic pro-cytokines.^21^ NLRP3-induced caspase-1 activation also activates nicotinamide adenine dinucleotide phosphate oxidase, playing a role in host defense.^22^The increase in NLRP3 expression in the 2-week-treated samples may be indicative of a quickened inflammatory and wound healing response. Wounds lacking NLRP3 exhibit reduced growth factor and macrophage infiltration.^23^ There is also a link between the activation of autophagy and NLRP3. Inflammasome activation triggers autophagy induction, and autophagy eliminates activated inflammasomes, which are important for immune homeostasis.^24^

The surgical procedure the patients in this study underwent results in the presence of debris that must be cleared for proper healing. Part of this debris includes lipid droplets. These large particles, which are released by adipocytes, are digested through autophagy (lipophagy), which alters these large lipid droplets into smaller products that can be taken up by macrophages, thus providing clearance.^25,26^ The treatment used in this study is designed to stimulate macrophage clearance, offering more efficient removal of lipid droplets, allowing for remodeling of the ECM.^2,7,27^ The gene expression patterns for the 2-week treatment clearly showed that treatment enhanced gene expression patterns related the initial immune response with an M1 macrophage stimulation and the transformation to an anti-inflammatory response, and the 4-week treatment, as described below, finalizes the story by shifting the gene expression pattern to reveal the activation of ECM remodeling.

There were only 2 upregulated genes in the 4-week-untreated group. Overall, the 4-week-untreated group appeared to have returned to the baseline gene expression. In contrast, the 4-week-treated group showed a unique pattern of upregulation. The upregulation of *NLRP3* observed in the 2-week-treated group was no longer seen in the 4-week-treated group. Long-term overexpression of *NLRP3* is associated with chronic, non-healing wounds, possibly due to the prevention of M2 macrophage polarization.^3,28^ A “switch” from M1 type macrophages to M2 is generally considered a sign of inflammation resolution and is associated with properly healing wounds. The data support that the upregulation of NLRP3 at week 2 was resolved by week 4. However, at 4 weeks, the treated group showed upregulation of NLRP12. NLRP12 is expressed primarily by myeloid-monocytic lineage cells, including monocytes, granulocytes, and eosinophils,^29^ functioning as a negative regulator of the inflammatory response.^30^ Taken together, the 4-week-treated group revealed a gene pattern that showed that pro-inflammation was diminishing, and anti-inflammation was in full force.

Furthermore, CD40LG was at the center of the 4-week-treated protein–protein interaction network, and it was linked to CCL22. This interaction is important for the recruitment of TH2 cells into inflammatory regions in order to regulate the TH-2-mediated immune response.^31^ M2 macrophages are the primary source of CCL22 production.^32^ This chemokine response was unique to the 4-week-treated group, further supporting activation of the anti-inflammatory response.

Finally, a decrease in fibrosis and/or skin hardening, which is known as induration was observed with the use of this product as measured by 2 clinical assessments. This was further supported by the gene expression data. For the 4-week-treated samples, matrix metalloproteinases 25 (*MMP25*) and transglutaminase (*TGM2*) were upregulated; these genes are associated with tissue remodeling. MMPs, including *MMP25*, are responsible for degrading ECM proteins, and this is an important part of wound healing.^33^ TGM2 is an important epidermal barrier protein that contributes to adhesion and epidermal–dermal integrity.^34^ Together, these data support that at 4 weeks, with the treatment, the gene expression pattern showed that ECM remodeling was activated, which was not observed in the 2-week-treated samples or in the 4-week-untreated group.

This study has limitations that should be addressed. First, while this is technically a randomized double-blinded study, the small sample size significantly undermines the power of the study. However, the data from the patients were consistent, statistically significant, and we did not observe any outliers, which suggests that the findings will hold up in a larger study in the future. In addition, we used Cetaphil as the bland moisturizer for the control. This was done to ensure that the effects we were observing were actually not just due to improved hydration of the area. To more accurately assess the effects of our treatments, we could have used the base material for the Alastin products. Finally, in order to verify our gene expression findings, in the future, we plan to explore protein expression as well, which will further provide evidence of the functional outcomes observed as a result of the treatment.

## CONCLUSIONS

For patients undergoing body procedures, a topical treatment of Alastin RSN and TFB Treatment with TriHex Technology induces an accelerated healing response that involves the clearance of “waste” products and the induction of anti-inflammatory genes. Furthermore, this topical treatment stimulates ECM remodeling, which ultimately improves the long-term results of the healing process, leading to less induration. A full clinical evaluation of this treatment and histological biopsy results will be presented in the near future in order to translate the gene expression data to patient outcomes.
